# Selecting elite groundnut (*Arachis hypogaea* L) genotypes for symbiotic N nutrition, water-use efficiency and pod yield at three field sites, using ^15^N and ^13^C natural abundance

**DOI:** 10.1007/s13199-017-0524-1

**Published:** 2017-11-20

**Authors:** Richard Oteng-Frimpong, Felix D. Dakora

**Affiliations:** 1 Department of Crop Sciences, Tshwane University of Technology, Private Bag X680, Pretoria, 0001 South Africa; 2 Savanna Agricultural Research Institute, CSIR, PO Box 52, Tamale, Ghana; 3 Chemistry Department, Tshwane University of Technology, Private Bag X680, Pretoria, 0001 South Africa

**Keywords:** N_2_ fixation, δ^13^C and δ^15^N values, %Ndfa, Multi-locational trials, N contribution, Symbiosis

## Abstract

About 70% of the groundnut (*Arachis hypogaea* L.) produced in Ghana is from the Guinea savanna. However, low soil nutrients, especially N, together with erratic rainfall distribution have often resulted in poor grain yield. The aim of this study was to evaluate plant growth, N_2_-fixing efficiency, N contribution, water-use efficiency and pod yield of 21 elite groundnut genotypes in the Guinea savanna of Ghana, using the ^15^N natural abundance technique. The data revealed significant variations in plant growth, symbiotic N contribution, and pod yield among the 21 genotypes tested at each field site. Average N contribution by groundnut genotypes ranged from 48 to 108 kg N ha^−1^. Also, mean pod yield ranged from 0.58 to 2.1 t ha^−1^. Genotypes ICGV-IS 08837, ICG 6222, ICGV 03315 and NKATIESARI demonstrated superior plant growth, symbiotic N contribution and greater pod yield. In fact, ICGV-IS 08837 yielded almost 2.5 fold more than CHINESE which is the most widely cultivated variety in the region. Genotypes ICGV-IS 08837, ICG 6222, ICGV 03315 and ICGV 99247 are therefore recommended for development into varieties for the Guinea savanna of Ghana. Genotypes ICG (FDRS) 4, ICGV00362 and ICGV99247 exhibited increased water-use efficiency, but were low in N_2_ fixation and N contribution, and would therefore be good parental material in breeding programs aimed at enhancing water-use efficiency in high N_2_-fixing genotypes.

## Introduction

Nitrogen (N) is an important component of plant growth and is needed for the biosynthesis of amino acids and proteins (Nelson et al. 2008) as well as chlorophyll for CO_2_ assimilation (Lawlor 2002). However, N is limiting in most agricultural areas around the world, in spite of its abundance as N_2_ in the atmosphere (Unkovich et al. 2008; Oldroyd et al. 2011). In Ghana, N and P are the most limiting plant nutrient in the Guinea savanna agro-ecology (Ahiabor et al. 2011). So, the ability of grain legumes to establish effective symbiosis with soil bacteria of the genera *Rhizobium* and *Bradyrhizobium* and fix atmospheric N_2_ provides legume species with an unlimited supply of symbiotic N (Unkovich et al. 2008; Oldroyd et al. 2011; Miransari et al. 2013; Vitousek et al. 2013). More specifically, groundnut is known to meet most of its N requirement from biological nitrogen fixation (BNF), while improving soil fertility (Nyemba and Dakora 2010; Mokgehle et al. 2014).

Groundnut is the most important grain legume in Ghana, and is largely cultivated in the Guinea savanna agro-ecology, which accounts for more than 70% of the national production (Tsigbey et al. 2003; MoFA-SRID 2014). This agro-ecology is characterised by acidic soils (pH 5–6.5), that are low in organic matter and N due to annual bush burning and crop residue removal (Abubakari et al. 2012). As a result, crop yields and productivity are low on farmers’ fields. The use of chemical N fertilisers can overcome N deficiency but they are expensive, inaccessible to smallholder farmers, and can potentially contribute to environmental pollution (Eickhout et al. 2006). The BNF is an important source of cheap and cleanly produced N for cropping systems and therefore a better alternative to the use of N fertilisers (Smil 1999; Nyemba and Dakora 2010; Mohale et al. 2014). Clearly, the inclusion of N_2_-fixing legumes such as groundnut offers a cost effective option for improving N availability in traditional cropping systems.

The amount of N-fixed in nodulated legumes is highly variable due to a range of factors including soil mineral N and the presence of adequate rhizobial numbers with high symbiotic efficacy (Abaidoo et al. 2007). The process is also constrained by other environmental and physiological conditions such as solar irradiance (Izaguirre-Mayoral and Sinclair 2009), drought (Pimratch et al. 2008a; Sinclair and Vadez 2012), soil temperature and deficiencies of P, iron (Fe), potassium (K), molybdenum (Mo) and manganese (Mn) (Izaguirre-Mayoral and Sinclair 2005; Vitousek et al. 2013; Divito and Sadras 2014). Soil water deficit and high temperature in the root zone hinder nodule establishment and nodule functioning (Liu et al. 2011), as well as the growth and survival of soil rhizobia (Miransari et al. 2013) which invariably affect the amount of N-fixed.

In Ghana, groundnut was reported to fix between 58 to 101 kg N ha^−1^ (Konlan et al. 2013), with an estimated benefit of 60 kg ha^−1^ fertiliser N to the succeeding maize crop in rotation (Dakora et al. 1987). In Zambia, BNF provided 70% of N to groundnut on farmers’ field and contributed between 19 to 79 kg N ha^−1^ to the cropping system (Nyemba and Dakora 2010). Studies by Pimratch et al. (2004) and Puangbut et al. (2011) in Taiwan have reported N contributions of 24 to 132 kg N ha^−1^ and 138 to 205 kg N ha^−1^ respectively, to the cropping system. In South Africa, groundnut was reported to contribute between 58 to 188 kg N ha^−1^ to the cropping system (Mokgehle et al. 2014). Therefore, N contribution by groundnut through BNF to the cropping system has the potential to improve soil N fertility and reduce the use of chemical N fertilisers, thus reducing the risk of eutrophication and hypoxia in water bodies, as well as global warming (Vance 2001; Miransari et al. 2013).

Several methods are currently used to estimate BNF in legumes in natural and agricultural ecosystems (Unkovich and Pate 2000; Unkovich et al. 2008). Of these methods, the ^15^N natural abundance technique has been used successfully to quantify N contribution in different legume species (Unkovich et al. 2008; Pule-Meulenberg et al. 2010; Belane et al. 2011; Mohale et al. 2014). In groundnut, the technique was used to measure the amount of N-fixed with high precision (Nyemba and Dakora 2010; Mokgehle et al. 2014). The method is based on the differences in ^15^N values between N_2_-fixing and non-N_2_-fixing species growing in the same soil, as well as on the assumption that the discrimination between ^14^N and ^15^N during soil N uptake and atmospheric N_2_ fixation is zero, or close to each other (Unkovich et al. 2008).

A few studies have assessed N_2_ fixation and N contribution by groundnut to cropping systems in Ghana (Dakora et al. 1987; Ennin et al. 2004; Konlan et al. 2013). Only a limited number of genotypes were tested using the N balance and/or N difference techniques. The aim of this study was to assess symbiotic N_2_ fixation in 21 groundnut genotypes in the Guinea savanna of Ghana, using the ^15^N natural abundance technique. Screening a large number of groundnut materials for symbiotic N nutrition could lead to the identification of high N_2_ fixing genotypes with greater growth and pod yield for use in breeding programs. Such genotypes have the potential to increase groundnut productivity in the Guinea savanna agro-ecology while improving soil fertility without employing N fertilisers.

## Materials and methods

### Experimental sites, groundnut genotypes and field setup

Field experiments were conducted at Nyankpala, Yendi and Damongo in the Guinea savanna of Ghana during the 2012 and 2013 cropping season. These sites have a unimodal annual rainfall between 900 and 1100 mm which starts from May and ends in September/October. The soils at these sites have a sandy loam texture and some mineral composition before planting are presented along with other environmental characteristics in Table 1.

**Table 1:** Description of environments used in this study

Environment	GPS coordinates	Total rainfall during trial	Maximum temperature	Total N	Organic C	Available P	Ca	S	pH
		(mm)	(°C)	(%)	(%)	(mg/kg)	(mg/kg)	(mg/kg)	
Nyankpala 2012	9.3913, −1.0025	519.8	30.9	0.03	0.42	0.7	290	1.18	4.92
Yendi 2012	9.4978, −1.0239	518.6	31.0	0.05	0.60	4.3	551	1.45	6.23
Damongo 2012	9.0447, −1.8144	373.5	31.9	0.03	0.37	8.7	278	2.30	5.37
Nyankpala 2013	9.3913, −1.0025	608.3	31.4	0.02	0.32	8.0	232	2.40	4.40
Yendi 2013	9.4959, −1.0222	539.8	31.3	0.03	0.50	7.0	432	2.00	5.50
Damongo 2013	9.0439, −1.8156	504.8	30.9	0.02	0.37	12.0	232	2.10	4.70

**Fig. 1:**
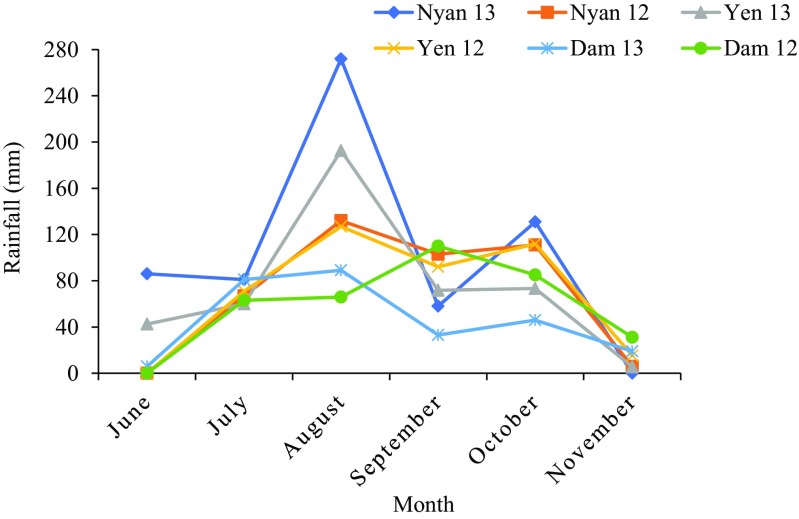
Monthly rainfall distribution in the Guinea savanna of Ghana in 2012 and 2013. Environment names are coded as Dam = Damongo, Nyan = Nyankpala and Yen = Yendi. The 12 refers to 2012 while 13 refer to 2013

**Fig. 2:**
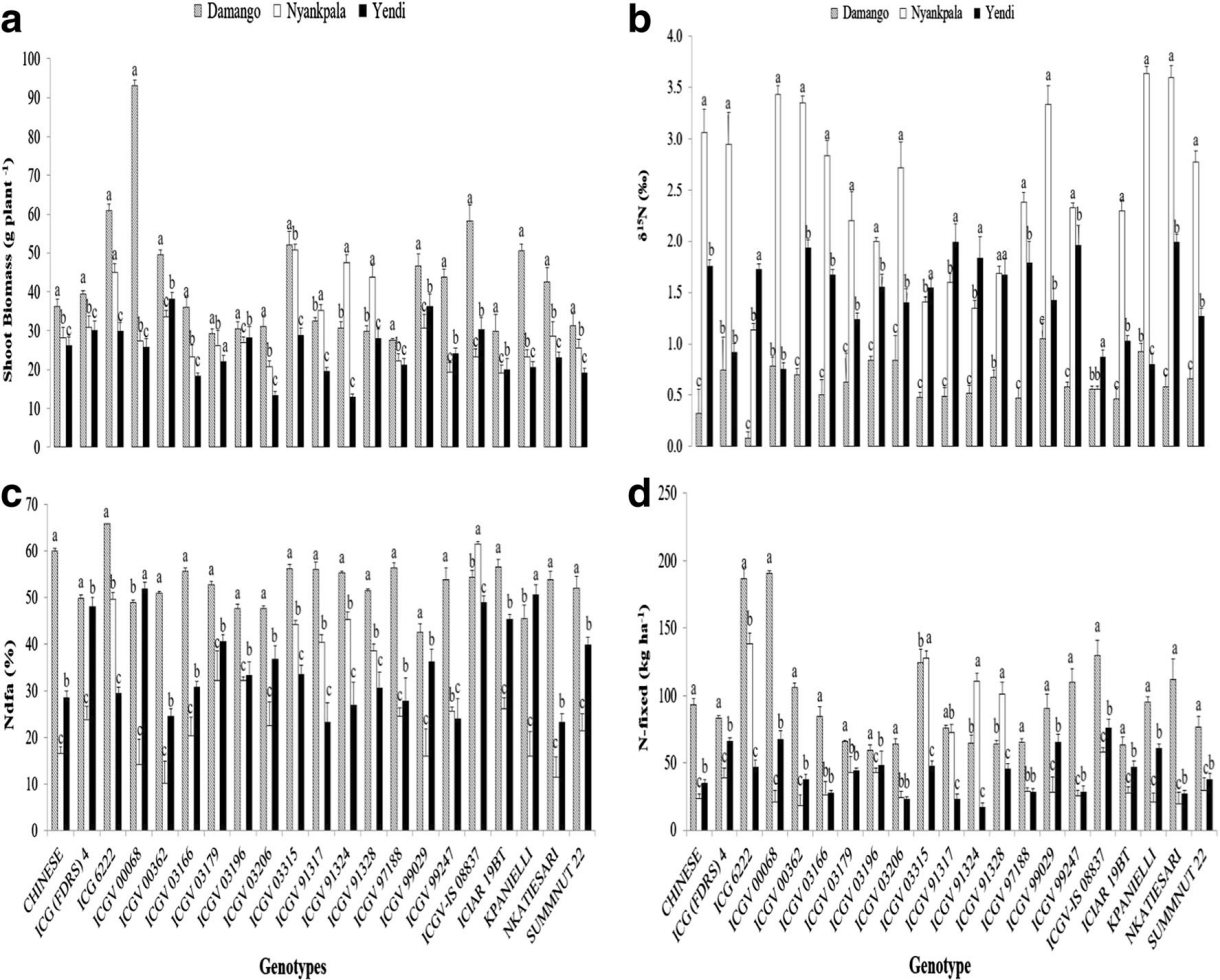
Interactive effect of genotype x environment on (**a**) Shoot biomass, (**b**) δ^15^N, (**c**) %Ndfa, and (**d**) N-fixed in 2012. Vertical lines on bars represent S.E. (*n* = 21). Bars followed by dissimilar letters are significantly different (*p* ≤ 0.05)

The groundnut genotypes used in this study and their sources are presented in Table 2. These genotypes exhibited different useful traits ranging from number of days to maturity, drought tolerance, foliar disease tolerance and tolerance to *Aspergillus flavus* infection. A randomised complete block design with four replicate plots for each genotype was employed. Each plot contained 6 rows and measured 3 m × 2 m. Groundnut genotypes were sown without rhizobium inoculation between June and July in both years. Inter-row and intra-row spacing was 40 cm and 15 cm respectively. There was no soil amendment and conditions of growth were similar to farmers’ practice in the region. Weeds were controlled manually with hand hoes on two occassions (Figs. 1 and 2).

**Table 2:** Genotypes used in this study and their sources

Genotype	Pedigree	Maturity class	Seed coat colour	Leaf colour score^#^	Source
CHINESE	Unknown	Early	Light tan	2	SARI, Ghana
ICG (FDRS) 4	ICGV 87157 (Argentine x PI 259747)	Late	Tan	3	ICRISAT, Mali
ICG 6222	Germplasm line	Late	Purple	5	ICRISAT, Mali
ICGV 00068	(ICGV 92069 x ICGV 94088) F2-SSD-SSD-B2-B1-B1(VB)	Late	Purple	3	ICRISAT, Mali
ICGV 00362	(ICGV 86300 x ICGV 92242)	Medium	Pale tan	3	ICRISAT, Mali
ICGV 03166	(ICGV 87378 x ICGV 96342)	Early	Pale tan	2	ICRISAT, Mali
ICGV 03179	(ICGV 96300 x ICGV 96352)	Early	Tan	2	ICRISAT, Mali
ICGV 03196	(ICGV 96342 x ICGV 98266)	Early	Tan	2	ICRISAT, Mali
ICGV 03206	(ICGV 98191 x ICGV 93382)	Early	Light tan	3	ICRISAT, Mali
ICGV 03315	(ICGV 91284 x ICGV 87846)	Early	Light tan	3	ICRISAT, Mali
ICGV 91317	(U4–7-5 x JL 24)	Early	Pale Tan	3	ICRISAT, Mali
ICGV 91324	(U4–7-5 x PI 337394F)	Early	Tan	2	ICRISAT, Mali
ICGV 91328	(J 11 x U4–7-5)	Early	Pale tan	3	ICRISAT, Mali
ICGV 97188	(ICGV 86887 x ICGV 87121)	Medium	Light tan	3	ICRISAT, Mali
ICGV 99029	(ICGV 94118 x ICGV 93427)	Late	Purple tan	4	ICRISAT, Mali
ICGV 99247	(ICGV 87354 x SANGDI)	Medium	Tan	3	ICRISAT, Mali
ICGV-IS 08837	(Agentine x PI29747) F3	Medium	Tan	3	ICRISAT, Mali
ICIAR 19 BT	ICGM/754 x ICGV 87922	Early	Light tan	3	ICRISAT, Mali
KPANIELLI	Unknown	Late	Red	5	ICRISAT, Mali
NKATIESARI	F-mix x ICG (FDRS) 20	Medium	Dark tan	5	SARI, Ghana
SUMNUT 22	Unknown	Medium	Dark tan	3	Nigeria

^#^Scoring done using the leaf colour chart (Witt et al. 2005)

### Plant sampling and processing

Five healthy plants were sampled from the middle rows 10 weeks after sowing. Harvested plants were packed individually into paper bags and oven dried at 60 °C for 72 h and weighed for dry matter determination. The shoot samples were then ground (0.50 mm sieve size) and stored prior to ^15^N analysis using mass spectrometry. Non-leguminous plant species (see Table 3) comprising both dicots and monocots were collected as reference plants from field plots and processed in a similar manner as the groundnut shoots for ^15^N analysis.

**Table 3:** Mean shoot δ^15^N values of non-legume reference plants used in calculating %Ndfa at each location in 2012 and 2013

Location	2012		2013	
	Species	δ^15^N (‰)	Species	δ^15^N (‰)
Nyankpala	*Celosia laxa*	+5.24	*Cassia obstusifolia*	+7.06
	*Euphobia heterophylla*	+3.96	*Dactyloctenium aegyptium*	+6.63
	*Hyptis suorelense*	+5.20	*Hyptis spp*	+4.05
	*Zea mays*	+4.63	*Sorghum bicolor*	+7.45
	Mean	+4.83 ± 0,30	Mean	+6.30 ± 0.77
Yendi	*Hyparrhenia inuolucrata*	+4.46	*Comelina spp.*	+3.34
	*Mitrocarpus villosus*	+3.22	*Panicum spp.*	+3.63
	*Vernonia ambigua*	+3.43	*Tridax procumbens*	+2.91
	*Zea mays*	+3.60	*Zea mays*	+2.76
	Mean	+3.58 ± 0.27	Mean	+3.16 ± 0.20
Damongo	*Acanthospermum hispidum*	+3.83	*Panicum spp*	+8.85
	*Andropogon gayanus*	+4.04	*Sorghum bicolor*	+8.77
	*Euphorbia heterophylla*	+3.71	*Tridax procumbens*	+5.90
	*Luduigia spp.*	+3.16	*Zea mays*	+5.01
	Mean	+3.69 ± 0.19	Mean	+7.09 ± 0.99

### Determination of shoot δ^15^N

Sub-samples of the grounded plant shoot were analysed at the Stable Light Isotope Laboratory, University of Cape Town South Africa, by combusting 2.0 mg ground powder in a Thermo 2000 Elemental Analyser coupled via a Thermo Conflo IV to a Thermo Delta V Plus stable light isotope mass spectrometer (Thermo Corporation, Bremen, Germany). An in-house reference material (*Nasturtium* spp.) was used as internal standard after every five sample runs to correct machine errors during isotopic fractionation. The ^15^N and ^14^N composition of each sample was read and the result was normalised and reported relative to N_2_ air. The isotopic deviation of ^15^N (δ^15^N) in the shoot of each sample was calculated as the difference in the atoms of ^15^N to ^14^N in the sample and the atmospheric N_2_ using the formula below (Mariotti et al. 1981; Pule-Meulenberg et al. 2010).

$$ {\updelta}^{15}\mathrm{N}\ \left({\mbox{\fontencoding{U}\fontfamily{wasy}\selectfont\char104}} \right)=\frac{{\left[{}{}^{15}\mathrm{N}/{}{}^{14}\mathrm{N}\right]}_{\mathrm{sample}}-{\left[{}{}^{15}\mathrm{N}/{}{}^{14}\mathrm{N}\right]}_{\mathrm{atm}}}{{\left[{}{}^{15}\mathrm{N}/{}{}^{14}\mathrm{N}\right]}_{\mathrm{atm}}}\kern0.5em \mathrm{x}\kern0.5em 1000 $$

### Percent N derived from the atmosphere (%Ndfa) and amount of N-fixed

The proportion of N derived from the atmosphere in each sample was calculated as (Shearer and Kohl 1986; Unkovich et al. 2008):

$$ \%\mathrm{Ndfa}=\left(\frac{\updelta^{15}{\mathrm{N}}_{\mathrm{ref}}-{\updelta}^{15}{\mathrm{N}}_{\mathrm{leg}}\ }{\updelta^{15}{\mathrm{N}}_{\mathrm{ref}}-{\mathrm{B}}_{\mathrm{value}}}\right)\ \mathrm{x}\kern0.5em 100 $$

Where *δ* ^*15*^ *N*_*ref*_ is the mean ^15^N natural abundance of non-legume species collected as reference plants from the experimental plots and processed as the groundnut shoot, *δ* ^*15*^ *N*_*leg*_ is the ^15^N natural abundance of each groundnut shoot, and the *B*_*value*_ is the ^15^N natural abundance of groundnut plants dependent solely on atmospheric N_2_ fixation for their N nutrition. For this study, the *B*_*value*_ used was −1.35‰ (Unkovich et al. 2008).

The amount of fixed-N in groundnut shoots was calculated as (Maskey et al. 2001; Pule-Meulenberg et al. 2010):

$$ \mathrm{N}\hbox{-} \mathrm{fixed}=\left(\frac{\%\mathrm{Ndfa}}{100}\right)\mathrm{x}\ \mathrm{shoot}\  \mathrm{biomass} $$

The amount of N-fixed was converted to kgNha^−1^ by multiplying the N-fixed per plant by groundnut plant population. Soil N uptake in shoots was calculated as the difference between total N and N-fixed.

### Statistical analysis

All data were tested for normality in distribution and then subjected to analysis of variance (ANOVA) using GenStat® Eleventh Edition. Where significant differences were found, the means were separated using the Duncan’s multiple range test.

## Location specific results using one-way ANOVA

### δ^15^N values of reference plants

At Nyankpala, the shoots of four non-legume species were sampled as reference plants during each cropping season (Table 3). In 2012, the highest δ^15^N (+5.24‰) was recorded in shoots of *Celosia laxa*, and the lowest (+3.96‰) in *Euphorbia heterophylla* providing a mean of +4.83‰ (Tables 3). In 2013, *Sorghum bicolor* showed the highest shoot δ^15^N (+7.45‰), and *Hyptis spp.* the lowest (+4.05‰).

At Yendi, four non-legume species were sampled as reference plants in both 2012 and 2013 (Table 3). *Hyparrhenia inuolucrata* showed the highest shoot δ^15^N in 2012 (+4.46‰), and *Blumea aurita* the lowest (+3.22‰), while in 2013, *Panicum* spp. recorded the highest shoot δ^15^N (+3.63‰) at Yendi and *Zea mays*, the lowest (2.76‰).

At Damongo, four non-legume species were sampled as reference plants in each cropping season (Table 3). In 2012, *Andropogon gayanus* revealed the highest shoot δ^15^N value (+4.04‰), and *Sporobolus* spp. the lowest (+1.97‰). In 2013, *Panicum* spp. recorded the highest shoot δ^15^N (8.85‰), and *Zea mays* the least (+5.01‰). Overall, shoot δ^15^N values were generally lower in 2012 compared to 2013 across the three locations. The minimum, maximum and mean δ^15^N values for each study site are shown in Table 4.

**Table 4:** Plant growth and symbiotic performance of 21 groundnut genotypes planted at Nyankpala in 2012 and 2013. Means followed by dissimilar letters are significantly different (*p* ≤ 0.05)

Genotype	2012									2013								
	Shoot biomass	Pod yield	N conc’n	N content	δ^15^N	Ndfa	N-fixed	Soil N uptake	δ^13^C	Shoot biomass	Pod yield	N conc’n	N content	δ^15^N	Ndfa	N-fixed	Soil N uptake	δ^13^C
	g plant^−1^	t ha^−1^	%	g plant^−1^	‰	%	kg ha^−1^	kg ha^−1^	‰	g plant^−1^	t ha^−1^	%	g plant^−1^	‰	%	kg ha^−1^	kg ha^−1^	‰
CHINESE	28bf	0.63bf	3.1c	0.9cg	3.06bd	29gh	42dg	103bg	−27.53de	28ad	1.12b	2.9 ac	0.8 ac	3.24bc	40hi	54 ac	82a	−27.77di
ICG (FDRS) 4	31bd	0.95b	3.1c	1.0ce	2.94 cd	31 g	50cf	110ae	−27.60dg	31 ac	0.80c	2.4ce	0.7ae	2.22j	53a	67a	58df	−27.42bg
ICG 6222	45a	0.85bd	3.7a	1.7ab	1.14j	60b	166a	112ad	−28.24i	23ce	0.13gh	2.6be	0.6eh	2.08j	55a	53ad	43 fg	−28.42jk
ICGV 00068	27bg	0.60bf	3.2bc	0.9cg	3.43ab	23hi	32 fg	113ad	−27.11bc	22de	0.22gh	3.1ab	0.6dh	3.06cf	42eh	43cf	59df	−27.47bh
ICGV 00362	34bc	0.79be	3.2bc	1.1c	3.35 ac	24hi	43dg	138a	−26.73ab	21de	0.20 gh	3.0ab	0.6dh	3.64a	35j	36ef	68ae	−27.33bf
ICGV 03166	23dg	0.53cf	3.1c	0.7dh	2.83d	32 g	39eg	83dh	−27.54de	26ae	0.04 h	2.1e	0.6eh	2.00j	56a	52bd	40 g	−27.71di
ICGV 03179	26cg	0.59bf	3.0c	0.9dh	2.20 g	43e	54ce	76eh	−27.62dg	32ab	0.64 cd	2.8ad	0.9a	2.85eh	45cf	65ab	80ab	−27.92fj
ICGV 03196	27cg	0.64bf	2.9c	0.8ch	2.00gh	46de	61 cd	72fh	−27.72eh	23ae	0.28eh	2.5be	0.8bg	3.00cg	43dh	48ce	63be	−27.75di
ICGV 03206	21 fg	0.47df	3.2bc	0.7eh	2.72df	34 fg	38eg	74fh	−28.16hi	24be	0.47df	2.7ad	0.6ch	3.01cg	43dh	46cf	61cf	−27.86ej
ICGV 03315	51a	0.51cf	3.4 ac	1.7a	1.41ij	55bc	160a	129ab	−27.99fi	19de	0.28eh	2.5be	0.5gh	3.36b	38i	32f	50eg	−27.27be
ICGV 91317	35b	0.42ef	3.1c	1.1c	1.60hi	52c	94bc	86ch	−28.04gi	26ae	0.04 h	2.4de	0.6ch	3.24bc	40hi	42cf	63be	−27.03ab
ICGV 91324	48a	0.42ef	3.1c	1.5b	1.35ij	56bc	137b	106af	−28.18i	24be	0.06 h	2.3de	0.5fh	2.96dg	44dg	40df	51eg	−27.18bd
ICGV 91328	44a	0.58bf	3.6ab	1.6ab	1.68 hi	51 cd	134b	130ab	−28.10hi	23ce	0.34eg	2.8ce	0.6eh	2.86eh	45cf	42cf	52eg	−27.07 ac
ICGV 97188	22eg	0.68bf	3.2bc	0.7eh	2.38eg	40ef	46cf	70gh	−27.26 cd	26ae	0.16gh	2.8ad	0.7af	2.77gi	46bd	55 ac	65ae	−27.70di
ICGV 99029	31be	0.90bc	3.2bc	1.0 cd	3.33 ac	24hi	39eg	127ab	−27.96ei	25ae	0.10gh	2.7ad	0.7bf	3.12 cd	42 gh	47ce	66ae	−28.08hk
ICGV 99247	19 g	0.35f	3.1c	0.6gh	2.32 fg	41e	41eg	59hi	−26.68a	18e	0.51de	2.6be	0.5 h	2.66 hi	48bc	36ef	40 g	−26.55a
ICGV-IS 08837	23dg	1.55a	2.5c	0.6 h	0.56 k	69a	65c	29i	−28.12hi	32ab	2.70a	2.4ce	0.8ad	2.60i	48b	62ab	67ae	−28.64 k
ICIAR 19BT	19 g	0.76be	3.2bc	0.6fh	2.29 fg	41e	42dg	60hi	−27.85ei	33a	1.19b	2.6be	0.9ab	2.82fi	45be	65ab	78 ac	−28.29ik
KPANIELLI	23dg	0.56cf	3.3bc	0.8dh	3.63a	19i	24 g	101bg	−27.61dg	21de	0.14gh	3.0ab	0.6ch	2.90dh	44cg	47ce	59df	−28.04gj
NKATIESARI	29bf	0.40ef	3.1c	0.9cf	3.59a	20i	30 fg	120 ac	−27.54de	25ae	1.11b	3.1a	0.8ad	3.10ce	42fh	55 ac	76ad	−27.89ej
SUMNUT 22	26cg	0.45df	3.1c	0.8dh	2.77de	33 g	44dg	88ch	−27.57df	26ae	0.24fh	2.8ad	0.7af	3.43ab	38ij	46ce	76ad	−27.67ci
s.e	5.01	165.2	0.27	0.18	0.29	4.2	12.2	20.8	0.27	3.63	107.8	0.23	0.08	0.11	2.0	8.7	10.9	0.26
CV (%)	16.6	35.9	8.5	18.3	12.0	10.6	18.5	22.0	1.00	20.4	29.8	12	17.2	5.2	4.5	18.1	17.6	1.3

### Plant growth

At Nyankpala, plant growth (measured as dry shoot biomass) varied significantly between and among the groundnut genotypes during the two cropping seasons (Table 5). The genotype ICGV 03315 showed the highest growth (50.9 g plant^−1^) in 2012, while ICIAR 19BT and ICGV 99247 recorded the least (19.1 and 19.4 g plant^−1^, respectively) in 2012. Other genotypes that produced high shoot biomass in 2012 included ICGV 91324, ICG 6222 and ICGV 91328 (47.6, 44.9 and 43.8 g plant^−1^, respectively). However, ICIAR 19BT revealed the highest growth (33.1 g plant^−1^) in 2013, followed by ICGV-IS 08837 (32.0 g plant^−1^), and the genotypes ICGV 03179 and ICG (FDRS)4 (31.6 and 30.6 g plant^−1^, respectively). In contrast, ICGV 99247 accumulated the least shoot biomass (17.9 g plant^−1^) in 2013.

**Table 5:** Plant growth, pod yield and symbiotic performance of 21 groundnut genotypes planted at Yendi in 2012 and 2013. Means followed by dissimilar letters are significantly different (*p* ≤ 0.05)

Genotype	2012								2013								
	Shoot biomass	N conc’n	N content	δ^15^N	Ndfa	N-fixed	Soil N uptake	δ^13^C	Shoot biomass	Pod yield	N conc’n	N content	δ^15^N	Ndfa	N-fixed	Soil N uptake	δ^13^C
	g plant^−1^	%	g plant^−1^	‰	%	kg ha^−1^	kg ha^−1^	‰	g plant^−1^	t ha^−1^	%	g plant^−1^	‰	%	kg ha^−1^	kg ha^−1^	‰
CHINESE	26be	2.8bc	0.7ch	1.76 ac	37gi	45eg	77bg	−27.51cf	20ch	0.47f	2.8ad	0.6bf	1.30i	41a	40ab	56gi	−27.33fh
ICG (FDRS) 4	30b	2.8bc	0.8bf	0.92eg	54ab	75 ac	64di	−26.77ab	20ci	0.81c	2.4df	0.5eh	1.69 g	33c	26dh	53hk	−26.54ab
ICG 6222	30b	3.2ab	1.0ab	1.72 ac	38gi	60ce	100ab	−27.97ef	32a	0.68de	2.8ad	0.9a	1.94ef	27e	40ab	109a	−27.44 gh
ICGV 00068	26bf	3.1b	0.8bg	0.75 g	57a	75 ac	57fj	−27.09 ac	23cf	0.46f	3.1a	0.7b	2.35b	18 h	21gj	95ab	−26.70bc
ICGV 00362	38a	2.4c	0.9ad	1.94ab	33hi	51ef	101ab	−26.48a	19di	0.43fh	2.5cf	0.5eh	2.19bd	22fh	17ik	63ei	−26.76bd
ICGV 03166	18gi	2.9b	0.5 hi	1.67 ac	39eh	35gi	55gj	−27.47cf	15i	0.16jk	2.3ef	0.3i	1.31i	41a	23ej	33 l	−27.42fh
ICGV 03179	22dg	3.0b	0.7eh	1.24df	48 cd	52df	57ej	−27.43cf	16gi	0.30hj	2.4df	0.4hi	1.55gh	36bc	22fj	40jl	−27.39fh
ICGV 03196	28bd	3.0b	0.8be	1.55bd	41eg	59de	82ad	−27.46cf	21cf	0.21ik	2.8ad	0.6bf	1.99de	26ef	25ei	72dg	−27.48gh
ICGV 03206	13 h	2.9b	0.4i	1.40 cd	44de	28hi	37j	−27.92df	18ei	0.16jk	2.6bf	0.5eh	1.73 fg	32 cd	25ei	54hj	−27.66 h
ICGV 03315	29bc	2.9b	0.8be	1.54bd	41eg	58de	83ad	−27.24bd	23ce	0.44 fg	2.2f	0.5dg	2.10ce	23eg	20hj	64dh	−26.76bd
ICGV 91317	19 fg	3.1ab	0.6gh	1.99a	32i	32 gi	68dh	−27.26be	19di	0.37fi	2.5cf	0.5fh	1.38hi	39ab	31ce	47il	−26.71bc
ICGV 91324	13i	3.0b	0.4i	1.83ab	35hi	22i	41ij	−27.47cf	21cf	0.10 k	2.7be	0.6cf	2.37b	18 h	17jk	78ce	−27.41fh
ICGV 91328	28bd	3.3ab	0.9ad	1.67 ac	39fh	58de	94 ac	−27.57cf	15hi	0.32 gj	2.7ae	0.4gi	1.19i	44a	30cf	38kl	−27.09cg
ICGV 97188	21eg	3.0b	0.6eh	1.79 ac	36gi	38fi	67dh	−27.43bf	21cf	0.25ik	2.9 ac	0.6bd	1.95ef	27de	28dg	75cf	−26.84e
ICGV 99029	36a	3.0b	1.1a	1.42 cd	44df	79ab	105a	−27.84df	25bc	0.30hj	2.7ad	0.7bc	2.72a	10i	11 k	104a	−27.39fh
ICGV 99247	24bg	3.0b	0.7ch	1.96a	33i	40fh	81ae	−26.46a	20di	0.08 k	3.0ab	0.6be	2.22bc	21gh	21gj	79 cd	−26.26a
ICGV-IS 08837	30b	3.1ab	0.9 ac	0.87 fg	55ab	86a	71ch	−27.44bf	21cg	1.36a	2.6be	0.5df	1.32i	41a	36bc	53hk	−27.20eg
ICIAR 19BT	20eg	3.1ab	0.6fh	1.03eg	52bc	53df	50hj	−27.84df	18fi	0.40fh	2.8ad	0.5dh	1.19i	44a	36bc	47il	−27.36fh
KPANIELLI	21eg	3.5a	0.7ch	0.80 g	56ab	68bd	53hj	−27.79df	24 cd	0.78 cd	2.6bf	0.6bd	1.71 g	32c	33bd	70dg	−27.22eh
NKATIESARI	23cg	3.1b	0.7dh	1.99a	32i	38fi	80bf	−27.94df	29ab	1.18b	2.8ad	0.8a	1.59gh	35bc	47a	89bc	−27.16dg
SUMNUT 22	19 gh	2.9b	0.6gi	1.27de	47 cd	44eg	50hj	−28.01f	22cf	0.65e	2.9 ac	0.6bd	1.24i	43a	44a	60fi	−26.98bf
s.e.	3.4	0.27	0.14	0.24	3.4	9.8	14.8	0.42	2.27	59.36	0.19	0.06	0.104	3.3	4.8	9.9	0.19
CV (%)	16.0	9.10	18.6	16.3	8.0	18.8	21.1	1.5	15.2	17.8	10.0	13.7	8.3	10.5	17.1	15.1	1.0

At Yendi, shoot biomass varied significantly (*p* < 0.001) among the genotypes in both cropping seasons (Table 6). The genotypes ICGV 00362, ICGV 99029, ICGV-IS 08837, ICG (FDRS) 4, and ICG 6222 produced the highest shoot dry matter (38.1, 36.4, 30.4, 30.0, and 29.8 g plant^−1^, respectively) in 2012 with ICGV 91324 producing the least dry matter (12.8 g plant^−1^) in 2012. However, genotypes ICG 6222, NKATIESARI, and ICGV 99029 produced the highest dry matter (32.3, 29.0 and 25.2 g plant^−1^, respectively) in 2013 while ICGV 03179 and ICGV 03166 yielded the least biomass (15.7 and 14.8 g plant^−1^, respectively).

**Table 6:** Plant growth, pod yield and symbiotic performance of 21 groundnut genotypes planted at Damongo in 2012 and 2013. Means followed by dissimilar letters are significantly different (*p* ≤ 0.05)

Genotype	2012									2013								
	Shoot biomass	Pod yield	N conc’n	N content	δ^15^N	Ndfa	N-fixed	Soil N uptake	δ^13^C	Shoot biomass	Pod yield	N conc’n	N content	δ^15^N	Ndfa	N-fixed	Soil N uptake	δ^13^C
	g plant^−1^	t ha^−1^	%	g plant^−1^	‰	%	kg ha^−1^	kg ha^−1^	‰	g plant^−1^	t ha^−1^	%	g plant^−1^	‰	%	kg ha^−1^	kg ha^−1^	‰
CHINESE	36 hj	1.2di	2.6ad	0.9bc	0.33i	67b	104dg	52 fg	−28.21cg	29f	0.7 h	2.0bc	0.6i	1.81eg	63 ac	59i	36 h	−27.60a
ICG (FDRS) 4	40 gi	1.6be	2.5bd	1.0b	0.75ce	58fh	97eh	69ef	−27.85bd	35cf	2.6a	2.3ab	0.8eg	1.58 fg	65ab	88 cd	47fh	−27.49a
ICG 6222	61b	2.8a	2.8 ac	1.7a	0.08i	72a	203a	80ce	−28.01bf	56a	2.4ab	2.5a	1.4a	2.50bd	54dg	128a	109a	−27.96a
ICGV 00068	93a	nd†	2.5bd	2.3a	0.79bd	58gi	224a	165a	−27.90be	38bd	2.5ab	2.4ab	0.9de	2.55 ac	54dg	79eg	69 cd	−27.80a
ICGV 00362	49df	nd	2.5bd	1.3a	0.70cf	59eh	123 cd	84be	−28.04bf	31df	0.9 h	2.5a	0.8eg	1.88eg	62 ac	82df	51fh	−27.56a
ICGV 03166	36 hj	1.1ei	2.5bd	0.9bc	0.50 gi	63bd	96ei	56 fg	−28.34eh	37be	2.4ab	2.0bc	0.7 fg	2.09cf	59bf	73fh	51fh	−27.93a
ICGV 03179	29jk	0.9fi	2.6ad	0.8bc	0.63dh	61cg	76hi	49 g	−28.32dh	30ef	1.5eg	2.5a	0.7 fg	2.22be	58cg	72gh	53eg	−27.81a
ICGV 03196	31jk	1.3ci	2.5 cd	0.8bc	0.84bc	57hi	71i	55 fg	−28.42fh	28f	1.8ce	2.3ab	0.6 hi	1.68eg	64 ac	68 h	38gh	−27.68a
ICGV 03206	31jk	0.9gi	2.6ad	0.8bc	0.84bc	57hi	76hi	58 fg	−28.70 h	30ef	1.3 g	2.3ab	0.8 fg	1.96cg	61ad	77eg	50fh	−27.46a
ICGV 03315	52 cd	2.0b	2.5ad	1.3a	0.48 hi	64bc	141bc	80ce	−27.69b	29f	2.0 cd	2.0bc	0.6i	2.27be	57cg	55i	41 gh	−27.75a
ICGV 91317	32ik	1.1ei	2.5bd	0.8bc	0.49 gi	63bd	86gi	50 g	−27.74bc	32df	2.6a	2.2 ac	0.7gh	1.78eg	63 ac	74fh	44 gh	−27.30a
ICGV 91324	31jk	1.4cg	2.3d	0.7c	0.52fh	63bd	74hi	44 g	−28.57gh	34cf	1.8de	2.5a	0.8df	1.96dg	61ae	84ce	54dg	−27.63a
ICGV 91328	30jk	1.4ch	2.5bd	0.8bc	0.68cg	60dh	75hi	50 fg	−28.42fh	42b	1.5eg	2.3ab	2.0bc	2.10cf	59bf	97b	67ce	−27.53a
ICGV 97188	27 k	1.8bc	2.5bd	0.7c	0.47hi	64bc	74hi	42 g	−28.31dh	34cf	1.4 fg	2.5a	0.8df	3.04a	48 h	68 h	74bc	−27.40a
ICGV 99029	47dg	nd	2.7 ac	1.3a	1.05a	52j	111df	102b	−28.53gh	40bc	1.8ce	1.9c	0.8 fg	1.85eg	62 ac	78eg	48fh	−27.98a
ICGV 99247	44eh	1.3ci	2.8 ac	1.2a	0.58eh	62cf	126bd	78de	−27.16a	31df	1.3 fg	2.4ab	0.7 fg	2.08cf	59bf	73fh	51fh	−27.67a
ICGV-IS 08837	58bc	nd	2.5 cd	1.4a	0.56eh	62cf	148b	90bd	−28.12bg	34cf	2.8a	2.3ab	0.8 fg	1.42 g	67a	85ce	42 gh	−27.81a
ICIAR 19BT	30jk	0.8hi	2.3d	0.7c	0.47hi	64bc	72hi	41 g	−28.25dh	40bc	1.4 fg	2.3ab	0.9 cd	1.99cg	61af	93bc	61cf	−27.82a
KPANIELLI	51de	1.4cf	2.5bd	1.3a	0.93ab	55ij	115de	97bc	−28.39fh	42b	1.7df	2.5a	1.0b	2.67ab	52gh	92bc	84b	−27.88a
NKATIESARI	42fh	1.7bd	2.9a	1.2a	0.58eh	62cf	128bd	79ce	−28.12bg	37be	1.9 cd	2.2 ac	0.8eg	2.06cf	60bf	79eg	54eg	−28.00a
SUMNUT 22	31jk	0.8i	2.8ab	0.9bc	0.66ch	60dh	88fi	58 fg	−28.26dh	33df	2.2bc	2.3ab	0.7 fg	2.15bf	59bg	73gh	51fh	−27.67a
s.e	4.84	37.72	0.21	0.15	0.12	2.3	15.6	11.8	0.29	2.93	164	0.16	0.0465	0.2462	2.917	3.81	6.62	0.36
CV (%)	11.5	20.7	8.3	14.2	18.7	3.7	14.2	16.8	1.0	11.7	12.6	10.0	8.1	16.8	6.9	6.7	16.8	1.8

^†^ *nd*, not determined

At Damongo, genotypes ICGV 00068 and ICG 6222 recorded the highest shoot biomass (92.9 and 61.0 g plant^−1^, respectively), and ICGV 97188 the lowest (27.5 g plant^−1^). Other genotypes that accumulated high shoot biomass included ICGV-IS 08837, ICGV 03315, and KPANIELLI with 58.2, 52.1, and 50.6 g plant^−1^, respectively (Table 7). However, genotype ICG 6222 produced the highest shoot biomass (56.3 g plant^−1^) in 2013, followed by ICGV 91324, KPANIELLI, and ICGV 99029 (42.5, 42.2, and 40.4 g plant^−1^, respectively). In contrast, ICGV 03196 recorded the lowest shoot biomass (27.8 g plant^−1^).

**Table 7:** A 2-Way ANOVA analysis of plant growth, pod yield and symbiotic performance of 21 groundnut genotypes planted at three locations in Ghana in 2012

Treatment	Shoot biomass	Pod yield	N conc’n	N content	Shoot δ^15^N	Ndfa	N-fixed	Shoot δ^13^C
	g plant^−1^	t ha^−1^	%	g plant^−1^	‰	%	kg ha^−1^	‰
Location								
Damongo	42.0 a	1.35 a	2.57 c	1.08 a	0.62 c	53 a	95 a	−28.16 c
Nyankpala	30.1 b	0.65 b	3.16 a	0.96 b	2.41 a	28 c	49 b	−27.67 b
Yendi	24.5 c	nd	3.01 b	0.73 c	1.48 b	35 b	43 c	−27.45 a
F-statistics								
Genotype	31.59***	10.21***	3.38***	26.44***	26.39***	15.00***	34.42***	14.20***
Location	306.13***	242.98***	122.51***	108.47***	1315.42***	468.56***	425.29***	103.35***
Genotype x Location	18.42***	5.76***	2.20***	13.90***	25.07***	13.34***	20.90***	2.69***

*ns*, not determined, *** - *p* < 0.001

### Pod yield

There was a substantial variation in pod yield between and among the genotypes at Nyankpala (Table 5). The highest yield in 2012 was by genotype ICGV-IS 08837 (1.55 t ha^−1^), followed by ICG (FDRS) 4 and ICGV 99029 (0.95 and 0.90 t ha^−1^, respectively), while ICGV 99247 produced the lowest (0.35 t ha^−1^). In 2013, the genotype ICGV-IS 08837 again recorded the highest pod yield (2.67 t ha^−1^), followed by ICIAR 19BT, CHINESE, and NKATIESARI (1.19, 1.12, and 1.11 t ha^−1^, respectively). In contrast, genotypes ICGV 91324 and ICGV 91317 recorded the least pod yield (0.06 and 0.04 t ha^−1^, respectively).

Although the pod yield was not determined at Yendi in 2012 due to logistical constraints, the 2013 data showed a high degree of variation between and among the genotypes (Table 6). Genotype ICGV-IS 08837 produced the highest pod yield (1.3 t ha^−1^), followed by NKATIESARI and ICG (FDRS) 4 (1.18 and 0.81 t ha^−1^, respectively), while genotypes ICGV 91324 and ICGV 99247 recorded the lowest pod yield (0.10 and 0.08 t ha^−1^, respectively).

At Damongo, pod yield was much higher in genotype ICG 6222 (2.85 t ha^−1^), followed by ICGV 03315 and ICGV 97188 (1.99 and 1.82 t ha^−1^, respectively), while SUMNUT 22 showed the least yield (0.8 t ha^−1^) (see Table 7). In 2013, however, ICGV-IS 08837 recorded the highest pod yield (2.8 t ha^−1^), followed by ICGV 91317 and ICG (FDRS) 4 (2.6 t ha^−1^ each), while CHINESE produced the least yield (0.7 t ha^−1^).

### Shoot δ^15^N values and %Ndfa

At Nyankpala, the δ^15^N of groundnut shoots and %Ndfa varied between and among the genotypes during both cropping seasons (Table 5). The genotypes KPANIELLI and NKATIESARI showed the highest shoot δ^15^N (+3.63‰ and +3.59‰, respectively), while ICGV-IS 08837 showed the lowest (+0.56‰) in 2012. As a result, ICGV-IS 08837 showed the highest %Ndfa (69%) while NKATIESARI and KPANIELLI recorded the lowest (20 and 19%, respectively). Other genotypes that obtained over 50% of their N nutrition from fixation included ICGV 91328, ICGV 03315, ICGV 91324, and ICGV 91317. In 2013, genotype ICGV 00362 displayed the highest shoot δ^15^N (+3.64‰), with ICGV 03166, ICG 6222 and ICG (FDRS) 4 being the lowest (2.00, 2.08 and 2.22‰, respectively). As a result ICGV 03166, ICG 6222 and ICG (FDRS) 4 met 56, 55 and 53%, respectively, of their N demand from fixation, while ICGV 00362 derived only 35% of its N nutrition from symbiosis.

At Yendi, shoot δ^15^N and %Ndfa values differed between and among the groundnut genotypes during the 2012 and 2013 cropping seasons (Table 6). Genotypes ICGV 91317, NKATIESARI, and ICGV 99247 recorded the highest shoot δ^15^N (+1.99, +1.99 and +1.96‰, respectively) in 2012, while ICGV 00068 and KPANIELLI showed the lowest (+0.75‰ and +0.80‰). As a result, ICGV 00068 and KPANIELLI derived the highest N from fixation (57 and 56%, respectively) and ICGV 99247, NKATIESARI, and ICGV 91317 the lowest (33, 32, and 32%, respectively). Genotypes ICGV-IS 08837, ICG (FDRS) 4 and ICIAR 19BT also obtained more than 50% of their N from fixation in 2012. However, genotype ICGV 99029 revealed the highest shoot δ^15^N (+2.72‰) in 2013, with ICIAR 19BT and ICGV 91328 as the lowest (+1.19‰). As to be expected, the genotypes with the lower δ^15^N values (ICGV 91328, ICIAR 19BT, SUMNUT 22, ICGV-IS 08837, ICGV 03166 and CHINESE) derived the most N from symbiotic fixation (44, 44, 43, 41, 41, and 41%, respectively), while ICGV 99209 which had the highest δ^15^N (+2.72‰), obtained the least N from fixation (10%).

At Damongo, shoot δ^15^N and %Ndfa differed significantly between and among the groundnut genotypes in both cropping seasons (Table 7). Genotype ICGV 99029 showed the highest shoot δ^15^N (+1.05‰) and ICG 6222 the lowest (+0.08‰) in 2012. As a result, genotype ICG 6222 recorded the highest %Ndfa (72%), followed by CHINESE (67%), with KPANIELLI and ICGV 99029 being the least (55 and 52%, respectively). In 2013, genotype ICGV 97188 displayed the highest shoot δ^15^N (+3.04‰), followed by KPANIELLI (+2.67‰), while ICGV-IS 08837 was the lowest (+1.42‰). As a result, ICGV-IS 08837 derived the highest N from fixation (67%), with ICGV 97188 and KPANIELLI being the lowest (48 and 52%, respectively).

### Amount of N-fixed

**Fig. 3:**
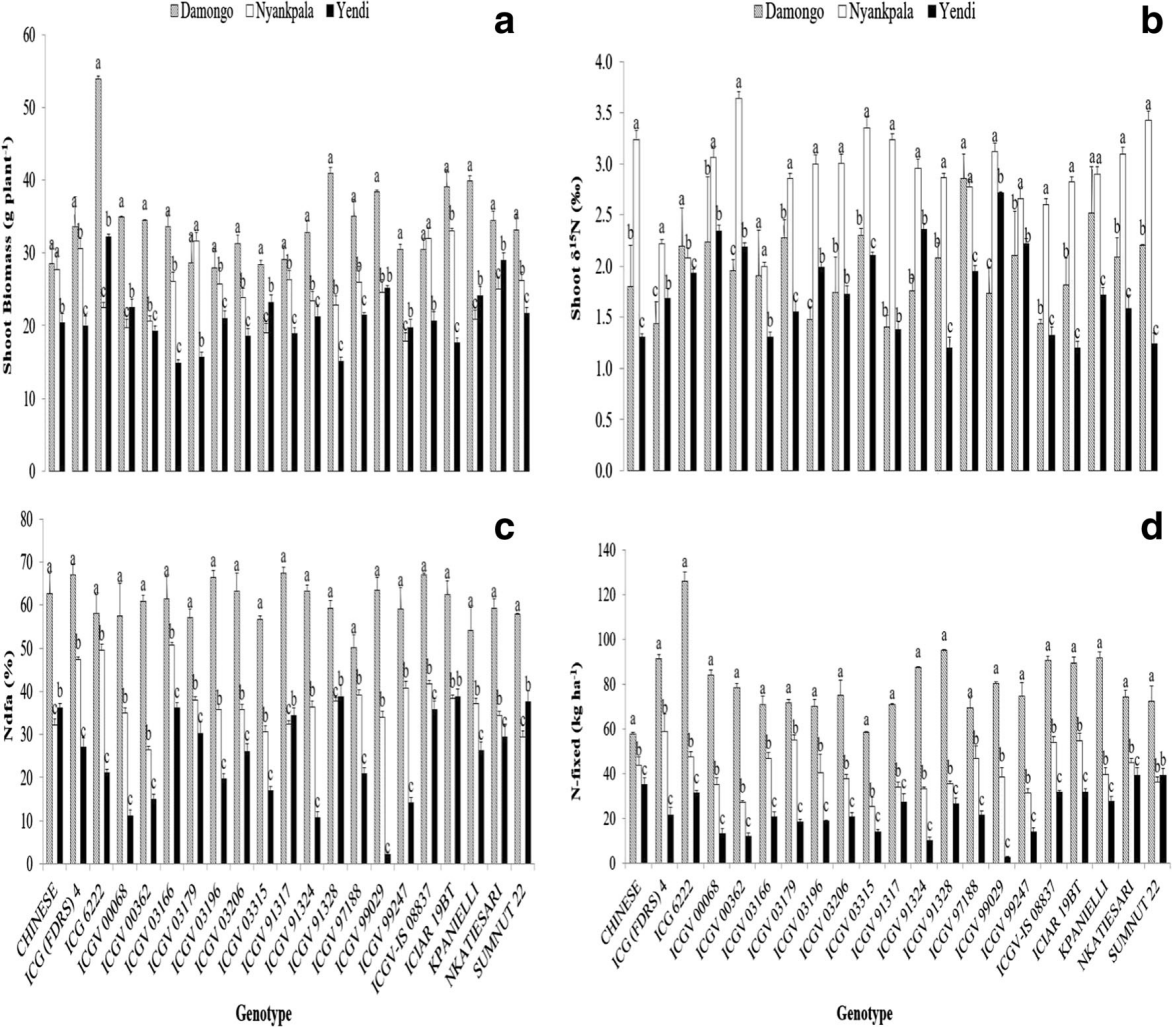
Interactive effect of genotype x environment on (**a**) Shoot biomass (g plant^−1^), (**b**) δ^15^N, (**c**) %Ndfa, and (**d**) N-fixed in 2013. Vertical lines on bars represent S.E. (*n* = 21). Bars followed by dissimilar letters are significantly different (*p* ≤ 0.05)

**Fig. 4:**
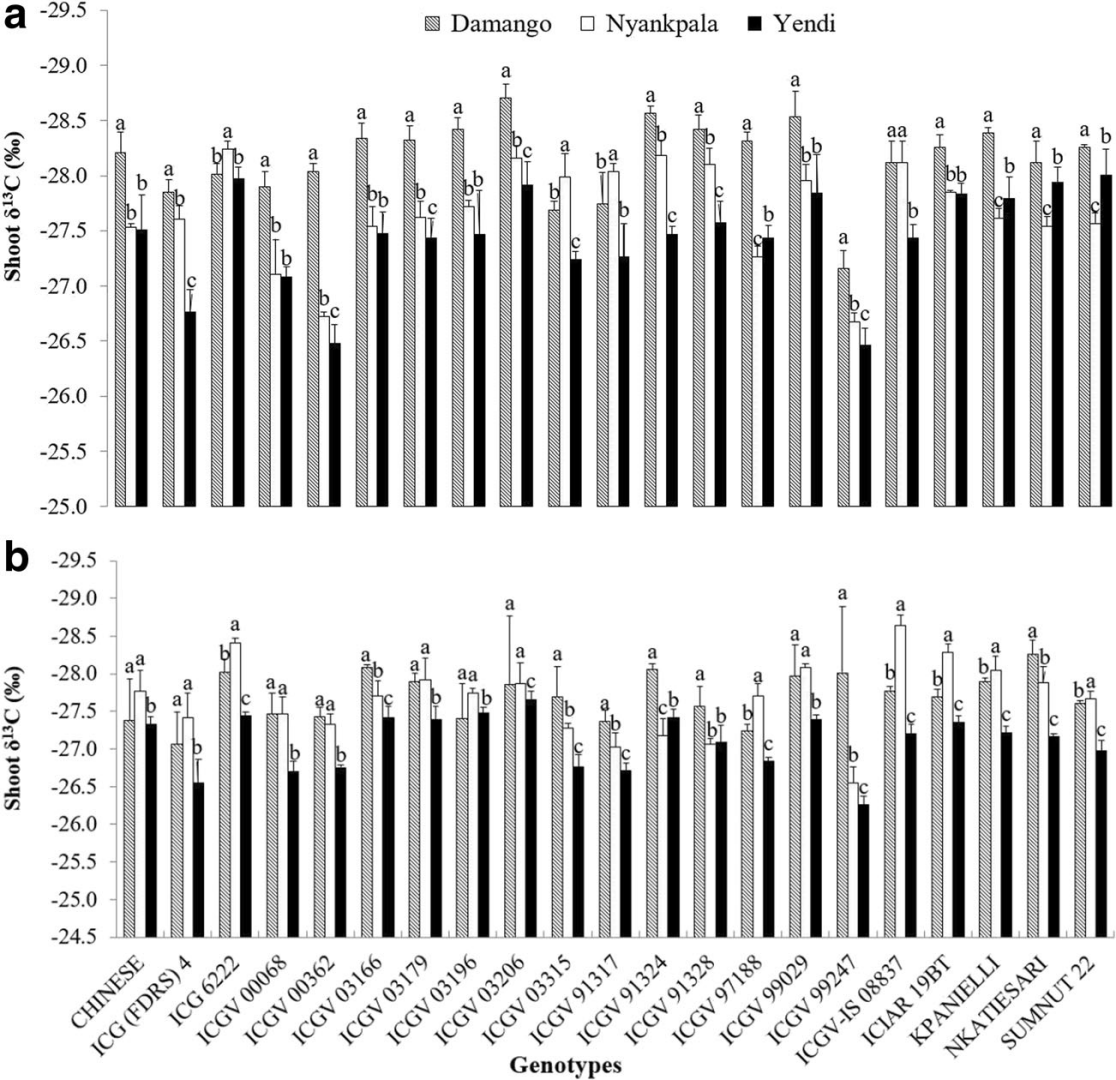
Interactive effect of genotype x environment on (**a**) Shoot δ^13^C in 2012, and (**b**) Shoot δ^13^C in 2013. Vertical lines on bars represent S.E. (*n* = 21). Bars followed by dissimilar letters are significantly different (*p* ≤ 0.05)

At Nyankpala, the amount of N-fixed was much higher in genotypes ICG 6222, ICGV 03315, ICGV 91324 and ICGV 91328 (166,160, 137, and 134 kg N ha^−1^, respectively) in 2012 and lowest in KPANIELLI (24 kg N ha^−1^) (Fig. 3a). In 2013 however, genotype ICG (FDRS) 4 contributed the highest amount of N (67 kg N ha^−1^), followed by ICGV 03179, ICIAR 19BT and ICGV-IS 08837 (65, 65 and 62 kg N ha^−1^, respectively), while ICGV 03315 was the least (32 kg N ha^−1^) (Fig. 4a). The amount of N-fixed in 2013 was generally lower than 2012 at Nyankpala.

At Yendi, genotype ICGV-IS 08837 contributed much more N (86 kg N ha^−1^) in 2012, followed by ICGV 99029, ICGV 00068 and ICG (FDRS) 4 (79, 75 and 75 kg N ha^−1^ respectively) (Fig. 3b). In contrast, ICGV 91324 yielded the least symbiotic N (22 kg N ha^−1^). However, in 2013, N contribution by NKATIESARI, SUMNUT 22, ICG 6222, and CHINESE was much higher (47, 44, 40, and 40 kg N ha^−1^ respectively), while ICGV 99029 produced the lowest (11 kg N ha^−1^) (Fig. 4b). The N contribution by groundnut was generally lower in 2013 than 2012 at Yendi.

At Damongo, symbiotic N contribution was greater in genotypes ICGV 00068 and ICG 6222 (224 and 203 kg N ha^−1^, respectively) in 2012. But other genotypes that also contributed substantial amounts of symbiotic N included ICGV-IS 08837, ICGV 03315, NKATIESARI, ICGV 99247, and ICGV 00362 (148, 141, 128, 126, and 123 kg N ha^−1^, respectively) (see Fig. 3c). Genotype ICGV 03196 produced the least symbiotic N (71 kg N ha^−1^) but N contribution by ICG 6222 was the highest (128 kg N ha^−1^) in 2013 (Fig. 4c), followed by ICGV 91328 and ICIAR 19BT (97 and 93 kg N ha^−1^, respectively), with ICGV 03315 recording the lowest N-fixed (55 kg N ha^−1^).

### Soil N uptake

Soil N uptake by groundnut genotypes was variable at Nyankpala in 2012 and 2013. The data showed that ICGV 00362, ICGV 91328 ICGV 03315 and ICGV 99029 took up the highest amount of soil N (138, 130, 129 and 127 kg N ha^−1^, respectively) in 2012, while ICGV-IS 08837 obtained the least (29 kg N ha^−1^). Genotype CHINESE also took up the highest amount of soil N (82 kg N ha^−1^) in 2013, followed by ICGV 03179 (80 kg N ha^−1^), with ICGV 99247 as the lowest (40 kg N ha^−1^).

At Yendi, ICGV 99029 took up more N from soil (105 kg N ha^−1^), followed by ICGV 00362 and ICG 6222 (101 and 100 kg N ha^−1^, respectively) in 2012. By contrast, ICGV 03206 obtained the least N amount of soil (37 kg N ha^−1^). But, ICG 6222 derived more N from soil in 2013 (109 kg N ha^−1^), followed by ICGV 99029 and ICGV 00068 (104 and 95 kg N ha^−1^), while genotype ICGV 03166 was the least (33 kg N ha^−1^).

At Damongo, genotypes ICGV 00068 and ICGV 99029 showed the highest soil N uptake (165 and 102 kg N ha^−1^, respectively) in 2012, followed by KPANIELLI (97 kg N ha^−1^), while ICIART 19BT was the least (41 kg N ha^−1^). In 2013, genotype ICG 6222 recorded the highest N uptake from soil (109 kg N ha^−1^), followed by ICGV 99029 and ICGV 00068 (84 and 74 kg N ha^−1^, respectively), while CHINESE was the lowest (36 kg N ha^−1^).

### Genotype × location interaction results

A two-way ANOVA analysis of genotype × location interaction revealed marked differences in plant performance between study sites (Figs. 2, 3 and 4). Groundnut shoot biomass was markedly greater at Damongo, (almost twice that of Yendi) and led to significantly increased pod yield when compared to Nyankpala. Shoot N content was higher at Damongo due to the greater shoot biomass. But shoot δ^15^N was lowest at Damongo, which resulted in higher N derived from fixation (53%) when compared to the much lower 28% obtained at Nyankpala (Fig. 2). This increased %Ndfa when combined with greater shoot biomass resulted in markedly larger amount of N-fixed (95 vs. 43 kg.ha^−1^ for Damongo and Yendi).

The genotype × location interaction was significant for all parameters tested in 2012 (i.e shoot dry mater, pod yield, N conc’n and content, shoot δ^15^N, %Ndfa, amount of N-fixed and shoot δ^13^C (Figs. 2 and 4). As shown in Fig. 3a, shoot dry matter was greater at Damongo, followed by Nyankpala, and lowest at Yendi. In fact, 17 out of 21 genotypes produced markedly larger shoot biomass at Damongo relative to Nyankpala and Yendi.

In contrast, shoot δ^15^N was significantly greater at Nyankpala in 2012. With 15 out of 21 genotypes recording the highest δ^15^N values, followed by five genotypes at Yendi (Fig. 2b). As a result, percent N derived from fixation was highest at Damongo and lowest at Nyankpala (Fig. 2c). Eighteen genotypes revealed the highest %Ndfa at Damongo relative to Nyankpala and Yendi. Symbiotic N contribution was therefore markedly greater at Damongo, with 18 genotypes producing the largest amount of N-fixed in 2012 (Fig.2d). The data obtained for 2013 were similar to those of 2012. Eighteen of the 21 genotypes tested produced the largest shoot biomass at Damongo (Fig. 3a). As found at Nyankpala in 2012, shoot δ^15^N was also markedly greater at Nyankpala compared to Damongo and Yendi (Fig. 3b). As a result, percent N derived from fixation was highest at Damongo and much lower at Yendi and Nyankpala (Fig. 3c). In fact, all 21 test genotypes obtained the most N from symbiosis, and therefore also contributed the largest amount of N at Damongo, followed by Nyankpala, and least at Yendi (Fig. 3d).

In general, shoot δ^13^C (a measure of water-use efficiency) was greater at Yendi in 2012, followed by Nyankpala, and lowest at Damongo (Fig. 4a). The data for 2013 showed a similar pattern to 2012, in that much higher δ^13^C values were recorded at Yendi.

### Correlation analysis

Correlation analysis showed a positive and significant relationship between shoot biomass and N content (*r* = 0.88, *p* < 0.001), N-fixed (*r* = 0.87 *p* < 0.001), pod yield (*r* = 0.56 *p* < 0.001) and %Ndfa (*r* = 0.50 *p* < 0.001). Soil N uptake was negatively correlated with %Ndfa. But pod yield was significantly correlated with %Ndfa and N-fixed.

## Discussion

Increased crop yields in the Guinea savanna of West Africa is constrained by low soil fertility (Cofie et al. 2005; Kombiok et al. 2005). The inclusion of N_2_-fixing legumes in cropping systems has the potential to overcome soil infertility and increase crop production (Sinclair and Vadez 2012). Dakora et al. (1987) have **shown that** cowpea and groundnut can make significant N contribution (201 and 101 kg N ha^−1^, respectively) to cropping systems in the Guinea savanna of Ghana and double the yield of a following maize crop. This has potential for increased food and nutritional security. In this study, 21 elite groundnut genotypes were assessed for N_2_ fixation, N contribution, grain yield and water-use efficiency using the ^15^N natural abundance. This ^15^N natural abundance method has been used to quantify N_2_ fixation in groundnut, and the data showed considerable variation in symbiotic N contribution (Nyemba and Dakora 2010; Mokgehle et al. 2014).

With the ^15^N natural abundance technique, quality data are obtained when the difference between the δ^15^N of reference plants and the test legumes is large, and at least equals to, or greater than, +2‰ (Unkovich et al. 1994). In this study, four reference plants were sampled per site and used to estimate soil N uptake by groundnut. Except for the Yendi site, where the difference between combined mean δ^15^N of reference plants and δ^15^N of groundnut was less than +2‰, at Damongo and Nyankpala this difference was generally above +2‰. Whether the low difference at Yendi was due to mychorrhizal infection of reference plants, which decreased the δ^15^N of the reference plants, was not assessed (Wheeler et al. 2000; Spriggs et al. 2003). However, the greater than +2‰ difference obtained between reference plants and groundnut at the other sites was considered high enough for a more precise measurement of N contribution by groundnut in Ghana’s Guinea savanna (Unkovich et al. 1994; Unkovich et al. 2008).

Location-specific differences were found between and among the 21 groundnut genotypes at all three study sites. For example, genotypes CHINESE, ICG (FDRS) 4, ICGV 03179, ICGV-IS 08837, ICIAR 19BT and NKATIESARI performed best at Nyankpala in 2013, while in 2012, ICG 6222, ICGV 91324, ICGV 91328, ICGV 03315 and ICGV 91317 ranked highest in amount of N-fixed, plant growth (shoot biomass), and pod yield (Table 5). At Yendi, genotypes ICG (FDRS) 4, ICG 6222, ICGV-IS 08837, ICGV 99029, ICGV 03315 and ICGV 91328 exhibited superior performance in 2012, while ICG 6222, ICGV-IS 08837, KPANIELLI, and NKATIESARI were the better performing genotypes in 2013 (Table 6). Similarly, at Damongo, the superior genotypes in 2012 included ICGV 00068, ICG 6222, ICGV-IS 08837, ICGV 03315, and KPANIELLI, while in 2013 genotypes ICG 6222, ICG (FDRS) 4, ICGV 91328, ICIAR 19BT, KPANIELLI and ICGV 99029 recorded much greater N_2_ fixation, which led to higher N content, greater shoot biomass and high pod yield (Table 7). The relatively superior performance of these genotypes was mainly due to their ability to fix higher amounts of N. In cowpea, greater N_2_ fixation led to the accumulation of other mineral elements in plant shoots, resulting in better plant growth and yield when compared to the low N_2_-fixing genotypes (Belane et al. 2014). This observation was confirmed by the positive correlation between N-fixed and pod yield in this study.

However, the strikingly different performance of the test genotypes at the same location over a two-year period could be attributed to soil nutrient imbalances between the two years and the different locations. For example, the 0.7 mg kg^−1^ available P in 2012 versus 8 mg kg^−1^ available P in 2013, 1.18 mg kg^−1^ S in 2012 against 2.4 mg kg^−1^ S in 2013, and the 290 mg kg^−1^ Ca in 2012 versus 232 mg kg^−1^ Ca in 2013 at Nyankpala (see Table 1) could potentially alter trait expression and normal growth of groundnut genotypes (Vitousek et al. 2013; Divito and Sadras 2014). Again, this huge differential in trait expression among genotypes could also be attributed to the large variation in rainfall distribution during field experimentation (see Fig. 1). Poor rainfall distribution coupled with low water-holding capacity of the soil due to their sandy texture could have exposed the groundnut to intermittent drought. For example, at Nyankpala, there was a dry spell after flowering which lasted for at least 25 days. This disparity in rainfall distribution could have altered N_2_ fixation, plant growth, and pod yield of the test genotypes (Serraj and Adu-Gyamfi 2009; Sinclair and Vadez 2012; Miransari et al. 2013; Abd-Alla et al. 2014).

Additionally, the symbiotic efficacy and population size of the rhizobia nodulating groundnut at each experimental site could have differed (Abaidoo et al. 2007; Pauferro et al. 2010). Studies of nodule occupancy with cowpea have shown that the variety Apagbaala, which was the second best N_2_-fixer of six genotypes when nodulated by only one strain (IGS type II), became the least N_2_- fixer of nine genotypes when nodulated by four strains (IGS types II, V, VIII and XVIII) (Pule-Meulenberg et al. 2010). So, independent of the experimental conditions at each site and the year of planting, these subtleties with nodule occupancy by rhizobia can potentially alter symbiotic N yield, and hence plant growth and pod yield.

At Nyankpala, only six and three out of the 21 genotypes in 2012 and 2013, respectively, derived over 50% of their N nutrition from symbiotic fixation. At Yendi, five out of 21 in 2012 and none in 2013 obtained over 50% of their N nutrition from fixation, while at Damongo all 21 genotypes met over 50% of their N demand from symbiosis in 2012, and 20 out of 21 in 2013. The greater dependency on N_2_ fixation by groundnut at Damongo relative to Nyankpala and Yendi could be attributed to endogenous concentrations of P and K (Vitousek et al. 2013; Divito and Sadras 2014). These nutrients were much higher at Damongo (8.7 mg kg^−1^ available P and 138 mg kg^−1^ K in 2012 or 12 mg kg^−1^ available P and 84 mg kg^−1^ K in 2013) when compared to Nyankpala with 0.7 mg kg^−1^ available P and 56 mg kg^−1^ K in 2012 or 8 mg kg^−1^ available P and 38 mg kg^−1^ K in 2013. Other studies similarly reported variation in symbiotic performance of groundnut planted at different locations (Pimratch et al. 2004; Pimratch et al. 2008a; Pimratch et al. 2008b; Mokgehle et al. 2014).

It was also interesting to note that only five out of the 21 groundnut varieties in 2012, and three out of 21 in 2013 obtained more N from fixation than from soil at Nyankpala. At Yendi, five out of 21 in 2012 and none in 2013 fixed more symbiotic N than they took up from soil, while at Damongo all the genotypes produced more N than they took up from soil in 2012 and only one genotype (ICGV 97188) obtained more N from soil than symbiosis. These results suggest that N_2_ fixation was generally inadequate in meeting the N demand, of groundnut genotypes at Nyankpala and Yendi. Whether the poor symbiotic performance was due to the ineffectiveness of the microsymbionts was not assessed (Abaidoo et al. 2007). Moreover, the concentration of N in soils was generally low at all experimental sites, and therefore less likely to have inhibited nodulation and N_2_ fixation (Ayisi et al. 2000; Ohyama et al. 2011; Tanabata and Ohyama 2014). Nonetheless, soil N uptake increased shoot δ^15^N values and resulted in less N derived from fixation.

Whatever the case, there were instances where some groundnut genotypes also contributed substantial amounts of symbiotic N and yet still took up large amounts of soil N. For example, at Damongo, genotype ICGV 00068 fixed 224 kg N ha^−1^ and took up 165 kg N ha^−1^, while genotype ICG 6222 fixed 128 kg N ha^−1^ and took 109 kg N ha^−1^ from the soil (Table 6). These findings suggest mineral N tolerance by those symbioses (Dakora 1998; Ayisi et al. 2000), a phenomenon that has potential for maintaining a positive soil N balance.

In both cropping seasons and across locations, shoot δ^15^N, %Ndfa and N-fixed were within the range of previous reports on groundnut symbiosis assessed using the ^15^N natural abundance technique (Nyemba and Dakora 2010; Rowland et al. 2012; Mokgehle et al. 2014). Groundnut is capable of obtaining 26 to 68% of it N requirement from symbiosis (Dakora et al. 1987; Phoomthaisong et al. 2003; Bado et al. 2006; Nyemba and Dakora 2010; Konlan et al. 2013; Mokgehle et al. 2014). The results of this study showed that groundnut can satisfy up to 72% of its N requirements from symbiosis with native rhizobia in the Guinea savanna and contribute up to 224 kg N ha^−1^. Other studies have reported that groundnut contributed 58 to 101 kg N ha^−1^ in the Guinea savanna (Dakora et al. 1987; Konlan et al. 2013) and 22–68 kg N ha^−1^ in the forest zone of Ghana (Konlan et al. 2013). Elsewhere, groundnut contributed 19 to 79 kg N ha^−1^ in Zambia (Nyemba and Dakora 2010), 44 to 247 kg N ha^−1^ in Thailand (Phoomthaisong et al. 2003; Pimratch et al. 2008a; Puangbut et al. 2011) and 58 to 188 kg N ha^−1^ in South Africa (Mokgehle et al. 2014). In this study, there was a strong relationship between N nutrition and plant growth, as well as pod yield. This was evidenced by the significant correlation between shoot biomass and %Ndfa (*r* = 0.50 *P* < 0.001), as well as N-fixed (*r* = 0.50 *P* < 0.001), just as pod yield was significantly correlated with %Ndfa (*r* = 0.63 *P* < 0.001) and N-fixed (*r* = 0.59 *P* < 0.001).

Given the erratic rainfall and its poor distribution in the Guinea savanna of West Africa, identifying groundnut varieties that exhibit improved plant water relations should be a first step to increasing grain yield. In this study, shoot δ^13^C values were found to differ between sites and even when grown in the same environment (Fig. 4). Groundnut plants sampled from Yendi showed much greater δ^13^C (or higher water-use efficiency), followed by Nyankpala, and then Damongo (Fig. 4). This suggests that the groundnut plants at Yendi were more water-use efficient in contrast to Damongo where they were less efficient. These findings are consistent with the rainfall distribution during the 2012 experimental season (Fig. 1). Whether in 2012 or 2013, groundnut shoot δ^13^C values showed the same pattern. Nine out of 21 genotypes exhibited much greater δ^13^C at Yendi in 2012 (Fig. 4a), while in 2013, 19 of the 21 genotypes recorded significantly higher δ^13^C values at Yendi when compared to Damongo or Nyankpala (Fig. 4b). Three groundnut genotypes (ICG (FDRS) 4, ICGV 00362 and ICGV 99247) consistently exhibited greater δ^13^C values at Yendi during both 2012 and 2013 cropping seasons (Fig. 4). Notwithstanding their greater water-use efficiency, the three genotypes were generally low in N_2_ fixation and N contribution (Table 6). Therefore, crossing them with high fixing and high yielding genotypes could produce progenies that are high yielding and water-use efficient.

Taken together, the 21 genotypes showed strong variation in symbiotic N dependency, N contribution, plant growth and pod yield. Genotypes ICGV 00068, ICG 6222, ICGV-IS 08837 and ICGV 03315 contributed the highest amount of symbiotic N, and also produced greater pod yield when compared to the most widely cultivated groundnut variety in the Guinea savanna. With further evaluation, these genotypes have a high potential to increase groundnut yield and productivity in the Guinea savanna. Although genotypes ICG (FDRS) 4, ICGV00362 and ICGV99247 exhibited increased water-use efficiency, they were low in N_2_ fixation and N contribution, and would be good parental material for breeding programs aimed at enhancing water-use efficiency in high N_2_-fixing genotypes.
